# Population genomics identifies the origin and signatures of selection of Korean weedy rice

**DOI:** 10.1111/pbi.12630

**Published:** 2016-09-30

**Authors:** Qiang He, Kyu‐Won Kim, Yong‐Jin Park

**Affiliations:** ^1^ Department of Plant Resources College of Industrial Science Kongju National University Yesan 32439 Korea; ^2^ Center for crop genetic resource and breeding (CCGRB) Kongju National University Cheonan 31080 Republic of Korea

**Keywords:** genetic resources, natural selection, origin, weedy rice, whole‐genome resequencing

## Abstract

Weedy rice is the same biological species as cultivated rice (*Oryza sativa*); it is also a noxious weed infesting rice fields worldwide. Its formation and population‐selective or ‐adaptive signatures are poorly understood. In this study, we investigated the phylogenetics, population structure and signatures of selection of Korean weedy rice by determining the whole genomes of 30 weedy rice, 30 landrace rice and ten wild rice samples. The phylogenetic tree and results of ancestry inference study clearly showed that the genetic distance of Korean weedy rice was far from the wild rice and near with cultivated rice. Furthermore, 537 genes showed evidence of recent positive or divergent selection, consistent with some adaptive traits. This study indicates that Korean weedy rice originated from hybridization of modern *indica*/*indica* or *japonica*/*japonica* rather than wild rice. Moreover, weedy rice is not only a notorious weed in rice fields, but also contains many untapped valuable traits or haplotypes that may be a useful genetic resource for improving cultivated rice.

## Introduction

Weedy rice (*Oryza sativa* f. *spontanea* Rosh.) is one of the most notorious weeds in rice fields worldwide, causing both crop yield losses and degrading the quality of rice (Qiu *et al*., 2014). It can be defined as any spontaneously and strongly shattering rice that occurs in cultivated rice fields, and it harbours phenotypes of both wild and domesticated rice. Weedy rice only occurs in or nearby rice fields and is characterized by its easy seed shattering, deep dormancy and red pericarp, which serve as keys to distinguish weedy rice from wild species and cultivated rice (Xia *et al*., 2011). Weedy rice produces fewer grains per plant and competes aggressively with the cultivated rice. It is present worldwide, including South and North America, southern Europe and southern and southeast Asia (Noldin *et al*., 1999; Sun *et al*., 2013). In the last 20 years, weedy rice has become a serious agricultural issue because of direct seeding. In southern parts of the United States, weedy rice causes an annual loss of more than $50 million (Gealy *et al*., 2002). In China, weedy rice affects more than three million ha and reduces the crop yield by about 3.4 billion kg (Liang and Qiang, 2011).

In Korea, a lot of weedy rice strains (*O. sativa* L., locally called ‘Aengmi’ and ‘Share’) have been collected from farmers’ fields. The regional distribution and genetics of weedy rice have been extensively characterized (Hak‐Soo and Mun‐Hue, 1992; Heu, 1988). ‘Share rice’ in Korea occurs mainly on Kanghwa Island and is geographically isolated from other weedy subpopulations. ‘Share rice’ has almost all of the characteristics of ‘Red rice’, while its spikelet within one panicle always has a different ripening time and the culm length is always shorter than those of ‘Red rice’ and landraces (Chung and Park, 2010). Although many morphological traits have been studied in detail, the large variability in complex quantitative traits in weedy rice remains unexploited (Chung and Park, 2010). Weedy rice plants are disease‐resistant and herbicide‐resistant (Chen *et al*., 2004; Olofsdotter *et al*., 2000). The seeds of weedy rice have high ability to persist in the soil (Delouche and Labrada, 2007). Therefore, it is difficult to obliterate weedy rice from rice fields. On the other hand, cultivated rice is susceptible to rice diseases such as rice blast disease, which leads to a 20%–30% loss of annual rice harvest (Kou and Wang, 2012). Weedy rice has attracted attention in the area of rice research because of its tolerance against biostress and abiostress (Chen *et al*., 2004; Liu *et al*., 2014; Lu *et al*., 2014; Qiu *et al*., 2014; Sun *et al*., 2013). Because weedy rice in Korea has acclimatized and adapted to different growing environments, it is also thought to be tolerant to a wide range of adverse conditions. In addition, we postulated that weedy rice may have additional useful characteristics, either in stress tolerance or rice eating quality, that could be used to improve cultivated rice in the future.

In addition, there is an active area of research toward determining the origin of weedy rice as a means to keep it out of rice fields. The origin of weedy rice has been debated intensively over the past 40 years, and there are three main hypotheses: (i) weedy rice descended directly from cultivated rice, with some populations from *indica*‐type cultivars (Londo and Schaal, 2007), some from *japonica*‐type cultivars (Cao *et al*., 2006, 2009; Vaughan *et al*., 2008), and others from *indica* × *japonica* hybrids (Ishikawa *et al*., 2005; Qiu *et al*., 2014); (ii) weedy rice may have generated from the process of ongoing selection and adaptation of wild rice (*Oryza rufipogon* and *Oryza nivara* which is considered as annual *O. rufipogon*) (De Wet and Harlan, 1975; Kelly Vaughan *et al*., 2001); and (iii) weedy rice may have originated from the hybridization between cultivated rice and its wild ancestor *O. rufipogon* (Londo and Schaal, 2007). A number of other hypotheses have also been proposed, including weedy rice originating from reversion of cultivated rice when domesticated rice was abandoned (Bres‐Patry *et al*., 2001) or forming by hybridization of its cultivated relatives (Ishikawa *et al*., 2005; Reagon *et al*., 2010; Xiong *et al*., 2012). This is supported by observations that some weedy‐type offspring could occur after inter‐subspecies and inter‐varietal hybridization in rice. Despite increasing focus on the evolutionary study of weedy rice, its origin is still unclear. A number of molecular markers have been applied to infer the population structure, genetic diversity and adaptive loci of weedy rice. However these molecular markers, either simple sequence repeats or restriction fragment length polymorphisms, which were frequently used in studies of weedy rice, only allowed scanning of the genome at very low density (Chung and Park, 2010; Gross *et al*., 2010; Kelly Vaughan *et al*., 2001; Londo and Schaal, 2007; Reagon *et al*., 2010; Sun *et al*., 2013; Thurber *et al*., 2010). It is easy to obtain biased conclusions with low‐density molecular markers because the accuracy of population structure, genetic diversity and adaptive loci identities is dependent on the representativeness of the markers, and it is difficult to evaluate the representative efficiency of the markers for unknown populations.

The rapid increase in the density of molecular markers through next‐generation sequencing (NGS) technology has facilitated building more solid hypotheses on genomics‐based study of rice (He *et al*., 2015; Huang *et al*., 2010, 2012a,b; Xu *et al*., 2012). Qiu *et al*. (2014) used a whole‐genome resequencing strategy for three weedy rice samples and suggested the origin of weedy rice from domesticated *indica/japonica* hybridization. However, the case study of these three weedy rice samples is not representative of all weedy rice populations, and the limited population size was not suitable to identify the adaptation loci for weedy rice.

Recently, a number of analytical methods have been performed to identify signals of recent positive selection or adaptation loci on a genome‐wide scale using huge NGS data, such as the fixation index (*F*_ST_) (Akey *et al*., 2002), nucleotide diversity (π) (Nei and Li, 1979), integrated haplotype score (iHS) (Voight *et al*., 2006), extended haplotype homozygosity (EHH) (Sabeti *et al*., 2002), cross‐population EHH (XP‐EHH) (Sabeti *et al*., 2007; Tang *et al*., 2007), composite likelihood ratio (CLR) (Nielsen *et al*., 2005; Williamson *et al*., 2007) and cross‐population CLR (XP‐CLR) (Chen *et al*., 2010). Most of these methods were first used in human populations and were later extended to animals and plants. All of the techniques significantly stimulate population genetic studies. In rice, Xu *et al*. (2012) detected many domesticated or selective sweeps by comparing the reduction in nucleotide diversity and *F*_ST_ between wild rice and cultivated rice. They found two well‐known domesticated rice genes, *prog1* (Jin *et al*., 2008) and *sh4* (Li *et al*., 2006), among their candidate selective sweep regions. However, this approach has not been used for weedy rice with NGS data.

In this study, we resequenced the whole genomes of 30 Korean weedy rice individuals and 30 Korean landrace rice individuals. Through high‐density single‐nucleotide polymorphism (SNP) markers and accredited population size, we performed population studies of Korean weedy rice to obtain reasonable hypotheses for the origin of Korean weedy rice. Furthermore, we detected many adaptive loci in the weedy rice population that may be valuable resources for control and utilization of weedy rice in the future.

## Results

### Polymorphisms across the rice genome

From high‐coverage whole‐genome sequencing of 30 weedy rice and 30 landrace unrelated individuals (Table S1) we collected more than two billion clean reads aligned to 374 Mb of the *O. sativa* genome (http://rapdb.dna.affrc.go.jp/download/archive/irgsp1/IRGSP-1.0_genome.fasta.gz). Ninety‐eight percent of reads were mapped to the reference with an average depth of 10× (Table S2). From these data, we detected more than eight million SNPs and one million InDels. For the ten wild rice plants we finally obtained 10.6 million SNPs and 1.3 million InDels (Table 1). It is reasonable that wild rice has much more variation than cultivated rice or weedy rice. In addition to SNP and Indel analysis, we studied the structure variations among different groups. We found 3957, 3754, and 3006 copy number variations among weedy rice, landrace rice and wild rice, respectively. A total of 19 391 inversions and 22 812 translocations were detected in weedy rice. While only 12 381 inversions and 19 767 translocations were detected in landrace rice (Table 1). It is suggested that weedy rice have much more structure variations than landrace rice.

**Table 1:** Summary of sequencing variations for 70 samples

Populations	Indels (m)	SNPs (m)	High‐quality Indels (m)	High‐quality SNPs (m)	CNV	Inversion	Translocation
Wild rice	1.33	10.63	0.914	6.96	3006	3666	24 357
Landrace rice	0.96	6.98	0.374	2.54	3754	12 381	19 767
Weedy rice	0.86	6.42	0.342	2.44	3957	19 391	22 812
Landrace_weedy	1.09	8.18	0.313	2.23	‐	‐	‐
Total	1.65	13.25	0.338	2.25	‐	‐	‐

CNV, copy number variation; SNP, single‐nucleotide polymorphism.

Most of the variations were rare (minor allele frequency [MAF] <0.05). To obtain the SNPs used for population studies, we excluded those missing in any of 70 accessions and those with an MAF of <0.05, because these variations may biased the population study. This yielded a final total of 2.2 million high‐quality SNPs (Tables 1 and S8). To our knowledge, this represents the largest high‐quality SNP data set for weedy rice reported to date. These data will be used to identify important adaptation loci of weedy or landrace rice, as well as for breeding. Indeed, using this data set we identified one novel allele on the *badh2* gene, which can be used to improve cultivated rice (He and Park, 2015).

### Population structure of weedy, landrace and wild rice

**Figure 1:**
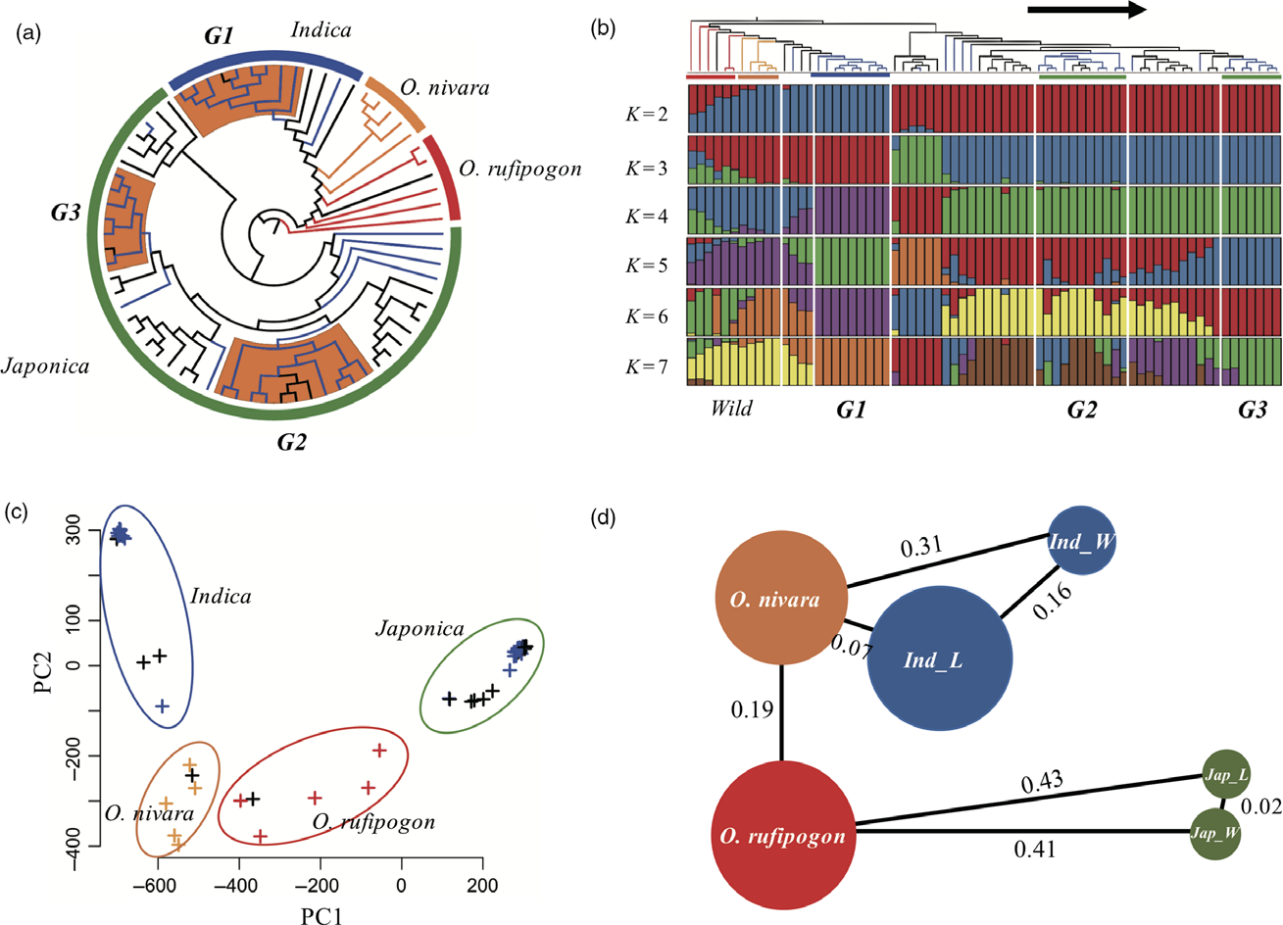
Population structure analysis of 30 weedy rice samples with 40 other *Oryza* species. (a) Maximum likelihood phylogenetic tree. Blue branches are weedy rice, black branches are landrace rice. (b) Maximum likelihood clustering with *K* ranging from 2 to 7. For each *K,* the different colour represents different populations. Each accession is represented by a vertical bar, and the length of each coloured segment in each vertical bar represents the proportion contributed by ancestral populations. (c) PCA plots. Blue dots are weedy rice, black dots are landrace rice. (d) Illustration of genetic diversity and population differentiation in wild rice and *japonica_landrace*,* japonica_weedy*,* indica_landrace* and *indica_weedy*. The sizes of the circles represent the levels of genetic diversity of groups, and the distances are *F*_ST_ values between different groups.

To understand the genetic population structure and relationships among the major groups of Korean landrace and weedy rice, we constructed a maximum likelihood (ML) tree and conducted population structure analysis based on the 2.25 million high‐quality SNPs. The ML tree contained four major groups corresponding to *O. rufipogon*,* O. nivara*,* O. japonica* and *O. indica*. This was consistent with the results of Xu *et al*. (2012). However, the *O. nivara* in our study was separated from cultivated rice compared with the results reported by Xu *et al*. (2012). This may have been because Korean *indica* rice has a different genetic background from those of other regions. The 30 weedy rice can be clearly divided into two major groups, *ind_weedy* (9) and *jap_weedy* (21). Similar to weedy, landrace can also be separated into two groups, *ind_landrace* (5) and *jap_landrace* (25). Moreover, we found that weedy rice could be divided into three major subgroups and some admixed individuals (Figure 1a). Because most people in Korea prefer *japonica* rice in their daily diet, it is reasonable that more than 76% of local rice samples belonged to the *japonica* group. Furthermore, we investigated the population structure of the 70 samples. We analysed the data by increasing *K* (number of populations) from 2 to 7 (Figure 1b). For *K *=* *2, we found a division between *japonica* and others. It is suggested the Korea *indica* rice was more closely related to wild rice than *japonica*. For *K *=* *4, *ind_weedy* (G1) was separated from *ind_landrace*. For *K *=* *5, one of the *jap_weedy* (G3) subgroups was clearly separated from *jap_landrace*, while another *jap_weedy* subgroup, G2, was always mixed with other *jap_landrace*. From *K *=* *2 to 7, there were no patterns indicating that weedy rice formed directly from wild rice. Because the phylogenetic tree showed that *ind_weedy* was consistently farther from wild rice than *ind_landrace* (Figure 1a), and it did not contain any wild patterns from the *japonica* rice group in the structural study (Figure 1b), we assumed that Korean *ind_weedy* rice was formed from *indica* rice without admixture of patterns from other populations. For *K *=* *5, the *japonica* group was separated into three subgroups, with many admixture patterns among the different subgroups. In comparison to *ind_weedy*,* jap_weedy* had more admixture patterns from among *jap_weedy* or between *jap_weedy* and *jap_landrace*, but not from *indica* or wild species. Therefore, we postulated that *jap_weedy* was derived from *japonica* rice and that gene exchange occurred frequently between or among *japonica* rice. Meanwhile, the PCA plot showed clearly that weedy rice was clustered with landrace rice in both the *indica* and *japonica* groups (Figure 1c).

For all subgroups, we calculated the π values with a 100‐kb sliding window, the common summary statistics for measuring genetics diversity in a population. The average diversity levels of *O. rufipogon* and *O. nivara* are markedly higher than those of *jap_landrace*,* jap_weedy*, and *ind_weedy*, but not *ind_landrace* (Figure 1d). It is suggested that Korean *japonica* rice may have undergone a stronger reduction in effective population size than *indica* or weedy rice, while the *indica* landrace may not have encountered a strong bottleneck during domestication. The *F*_ST_ by the 100‐kb sliding window was used to measure the level of population differentiation. The average *F*_ST_ between *O. rufipogon* and *O. nivara* (0.19) was much lower than that between wild rice and *O. sativa* [*ruf‐jap_weedy* (0.41), *ruf‐jap_landrace* (0.43), *niv‐ind_weedy* (0.31). However, the *F*_ST_ between *O. nivara* and *ind_landrace* was only 0.07, which was much lower than that with *ind_landrace‐ind_weedy* (0.16). This suggests that *indica* landrace is more closely related to *O. nivara* than the local weedy rice, possibly due to the materials of *O. nivara*. In the study by Xu *et al*. (2012), these five individuals were mixed with the *indica* varieties in the phylogenetic analysis, like in our study. The average *F*_ST_ between *jap_landrace* and *jap_weedy* was only 0.02 because of the complex and frequent gene exchange between these two subgroups (Figure 1d). This was confirmed by phylogenetic and population structure pattern analysis.

To estimate the linkage disequilibrium (LD) patterns in different rice groups, we calculated *r* ^2^ between pairs of SNPs using PLINK. We quantified the average extent of the genome‐wide LD decay distance in wild rice, Korean landrace rice, and Korean weedy rice. These estimates were approximately 11, 72 and 80 kb in these three groups, respectively, where the *r* ^2^ dropped to half from the highest value (0.50, 0.53 and 0.66 respectively) (Figure S1). The LD decay of wild rice here was the same as reported previously. Because our weedy and landrace groups included *indica* and *japonica* subgroups, the LD decay was lower than *japonica* and larger than *indica*, as expected.

### Recent positive and divergent selection

**Figure 2:**
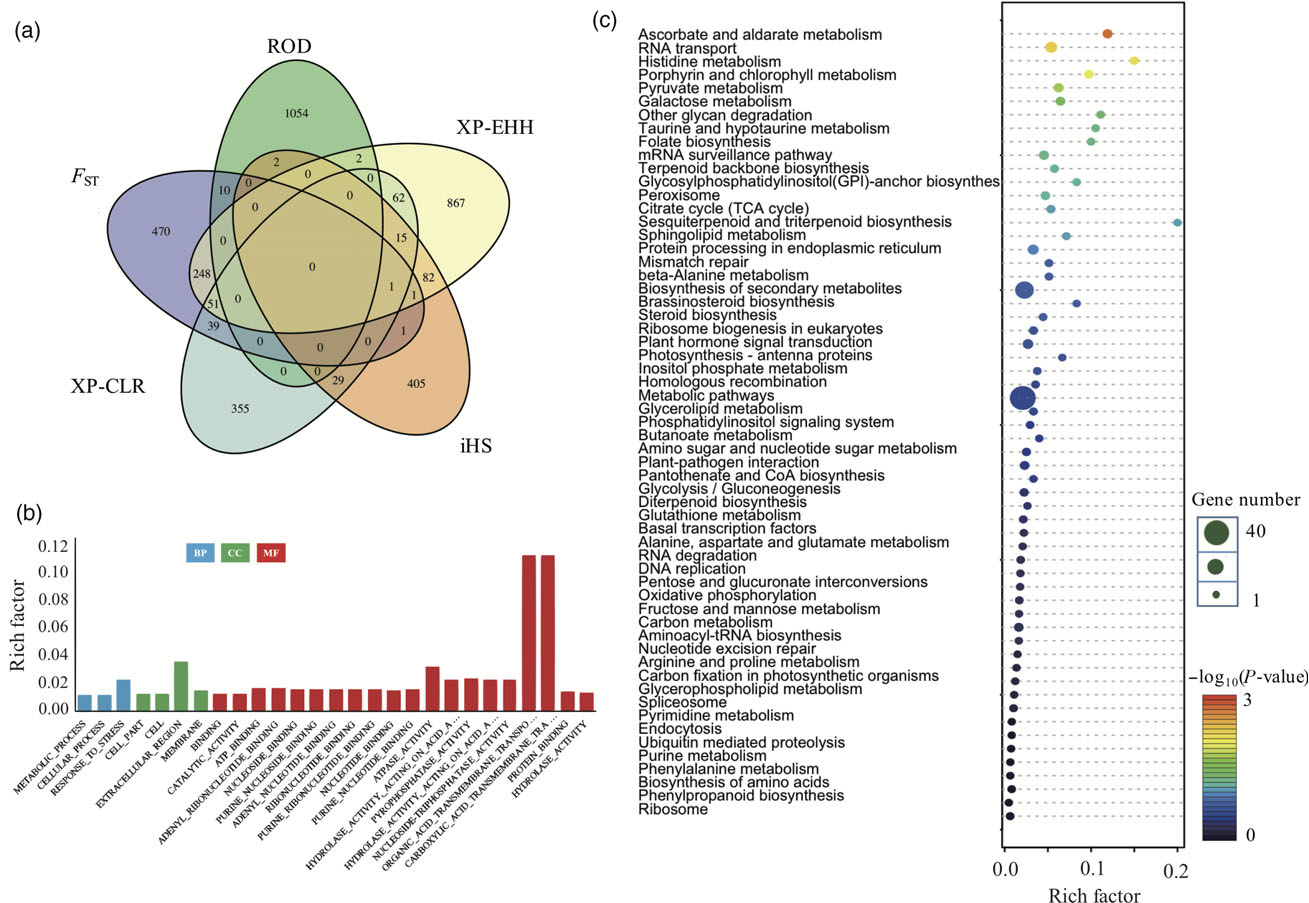
Unique and shared genomic regions (genes) among five selection scans. (a) Venn diagram of the number of genes throughout the genome in the top 2.5% for each selection scan. (b) GO enrichment of candidate selection genes. The blue bar indicates candidate selection genes, and the green bar shows the rice reference genome genes (c) KEGG pathway enrichment of candidate selection genes.

Population studies suggested that weedy rice formed from modern rice and not wild rice. LD patterns showed large differences in genome patterns between landrace and weedy rice, although the population study suggested that landrace rice and weedy rice undergo frequent gene exchange. To find the adaptive genome patterns of weedy rice, we employed five different methods to detect selective signatures between Korean landrace rice and weedy rice. Five distinct metrics of natural selection using a 100‐kb sliding window or per site across the whole genome were used; that is, differentiation (*F*_ST_), reduction in diversity (ROD), XP‐CLR, iHS and XP‐EHH. From these data, we classified the empirical top 2.5% of the windows or regions as ‘selection outliers’. After annotation, we found 1068, 821, 552, 536 and 1329 genes over the outliers using ROD, *F*_ST_, XP‐EHH, iHS and XP‐CLR respectively (Table S3). Most of the significantly selected genes (top 2.5%) occurred only one time among different selection approaches, suggesting that each statistic method will give a different view of selection. It is similar with previous study on *Populus trichocarpa*, that different selective forces are shaping different genomic regions (Evans *et al*., 2014). We also termed the regions in the top 2.5% for at least two of the selection scan metrics as candidate selection genes. Finally, 537 genes were detected as candidate selection genes (Table S4 and Figure 2a).

To explore the biological functions of 537 candidate selective genes, we performed a functional enrichment analysis to identify gene ontology terms. In total, 27 significantly enriched GO terms (*Q *<* *0.05) were characterized in three major categories: biological process, cellular component and molecular function (Figure 2b and Table S5). By using the rice GO terms as background, the candidate selected genes were enriched in response to stress, metabolic process and cellular process in biological process; extracellular region, membrane, cell and cell part in cellular component; organic acid transmembrane transporter activity, carboxylic acid transmembrane transporter activity and other 18 categories in molecular function (Figure 2b). Meanwhile, we performed KEGG pathway analysis for these 537 genes, which were enriched into 59 pathways, especially ascorbate and aldarate metabolism, RNA transport, histidine metabolism and Porphyrin and chlorophyII metabolism pathways (Figure 2c and Table S6).

**Figure 3:**
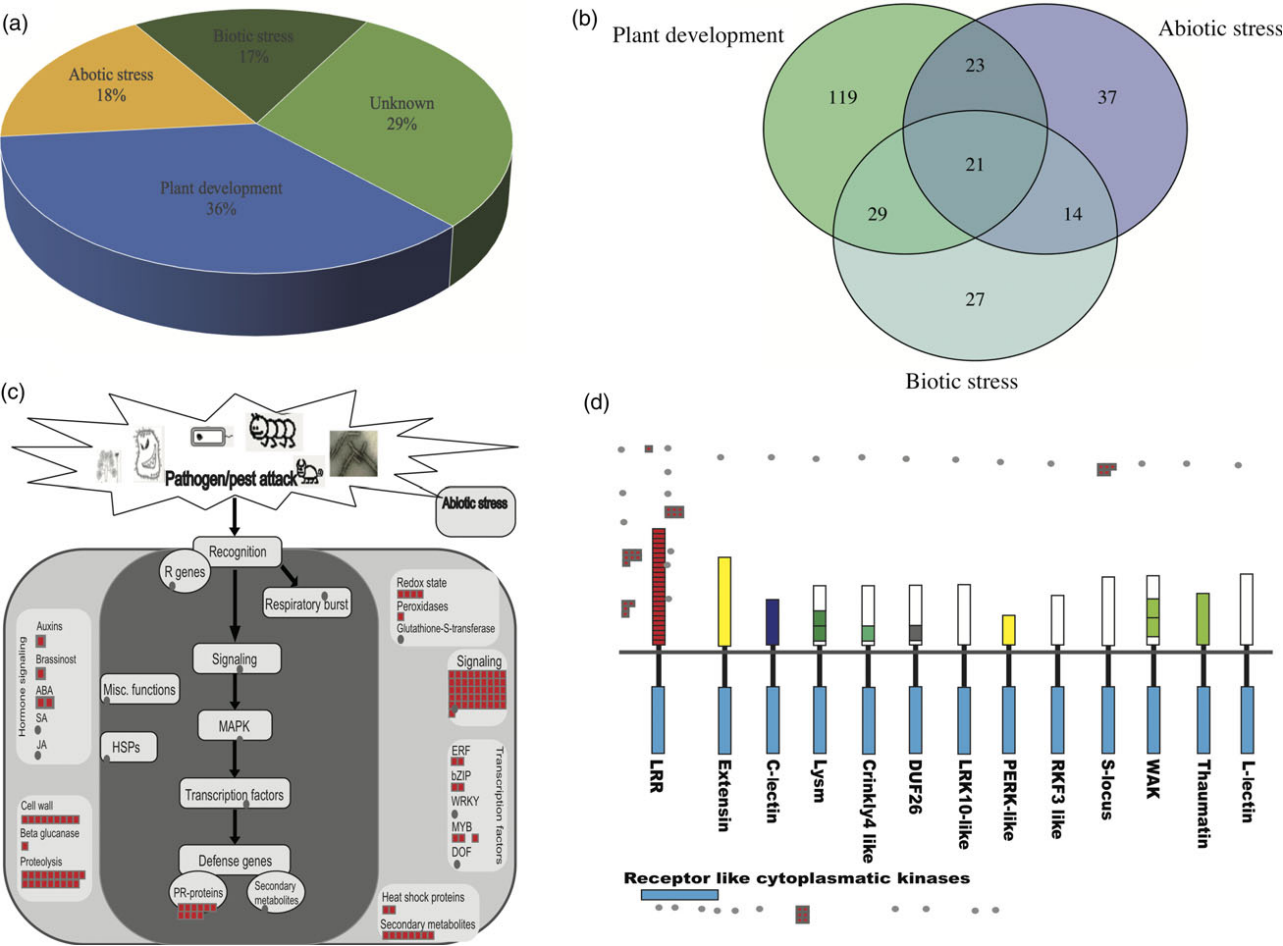
Candidate selected genes clustered based on public databases. (a) Functional category of candidate selected genes. (b) Venn diagram of three functional category. (c) The ‘Biotic Stress overiew’ MapMan pathway was used to visualize candidate genes. Red square are genes. (d) Genes involved in receptor‐like kinases. Red square are genes.

Furthermore, we clustered those candidate selective genes against published database by using the PantGSEA. All genes were clustered into 90 independent experiment based data sets. We clustered these experiment‐based data sets into three categories: plant development or growth‐related categories, which are concentrated in tissue‐specific expression genes; abiotic stress‐related categories, including genes regulated by abiotic stress, such as cold stress, drought stress, salinity stress, Pi starvation stress, Cr stress, Fe stress, auxin stress etc.; and biotic stress‐related categories, which were regulated by agrobacterium infection, blast fungus, bacterium infection in rice (Table S7). Among the 537 genes, 36% were related to plant development, 18% were related to abiotic stress, 17% were related to biotic stress and the remaining were still unknown in current rice database (Figure 3a). Some genes played different roles in different biological processes, in which 21 genes were clustered into three categories, and 66 genes shared in two different categories (Figure 3b).

To check the RNA expression pattern in specific tissues, we performed *k*‐means co‐expression clustering for these candidate selection genes. We downloaded the expression patterns of 11 different rice tissues from RiceXPro database (http://ricexpro.dna.affrc.go.jp). Three hundred and ninety‐seven candidate selection genes were detected among this database. We clustered ten co‐expression clusters (Figure S2 and Table S9) across all 11 tissues. Genes were defined as tissue‐specific expressed genes if their tissue expression pattern deviation over twofold of standard deviation among the cluster. Cluster 10 was removed from the tissue‐specific expression genes due to very low expression level. Finally, we found six tissue‐specific expression clusters (cluster 1, 2, 3, 4, 8, 9) among the ten clusters (Figure S2). Cluster 1, 4, 8, 9 have high expression patterns in rice leaf, endosperm, embryo and root respectively. We found two pleiotropic drug resistance (PDR) family genes in cluster 9, *PDR*8 and *PDR*9 (Table S9). The *PDR*9 in rice were significantly induced by jasmonates, which are involved in plant defence (Moons, 2008). We also found two receptor‐like cytoplasmic kinase (RLCK) family gene cluster 1, which play roles in development and stress response in plants (Vij *et al*., 2008). It is suggested that genes in cluster 1, 4, 8, 9 may play important roles in weedy rice development and stress response. Cluster 2 and 3 show low expression levels in endosperm and leaf respectively. These genes in cluster 2 and 3 may not play important roles for rice endosperm and leaf development, but important for other rice tissue development.

In order to obtain a global view of gene functions in biotic stress, we used MapMan analysis, which groups candidate gene sets into hierarchical functional categories on the basis of putative involvement in biotic stress (Figure 3c). Four hundred and forty‐eight genes out of 537 candidate genes were mapped to MapMan rice database. Among that, 107 genes were mapped to biotic stress pathway. Including five genes related to respiratory burst, 46 genes related to signalling, seven are transcription factors, eight secondary metabolites, two heat shock proteins, ten PR‐proteins, five related to hormone signalling, nine cell wall genes, 19 proteolysis related genes and one beta glucanase. These annotations provided a valuable source of data for investigation of processes, function and pathways involved in the reaction to biotic stress in weedy rice. When we preformed the same strategy to overview the receptor‐like kinases, 29 genes were enriched. Most of them (18) have LRR motifs, which were usually involved in protein–protein interactions and played an important role in regulating plant development and defence (Diévart and Clark, 2004). Six genes are receptor‐like cytoplasmatic kinases, which may related to rice development or stress (Vij *et al*., 2008). Five genes are S‐locus genes, which related to self‐incompatibility responses of crucifers (Xing *et al*., 2013) (Figure 3d).

## Discussion

Weedy rice is a noxious agriculture pest that has significantly reduced the grain yield of cultivated rice worldwide (He *et al*., 2014; Qiu *et al*., 2014). However, it has recently been realized that weedy rice possesses a number of adaptive traits lacking in cultivated rice, such as high reproduction and both biostress and abiostress tolerance (Green *et al*., 2001), so it may become an important resource for improving current cultivated rice. Knowledge regarding the origin, genetic diversity, structure and adaptive genomic loci of weedy rice populations will facilitate the design of effective methods to control and use this weed.

### Origin of Korean weedy rice

Most previous population genetics studies of weedy rice used limited polymorphism markers. Here, we used the whole‐genome resequencing method and detected more than two million high‐quality SNPs from 30 weedy rice, 30 landrace rice and ten wild rice samples. Using these high‐density SNP makers (approximately 5.2 SNP/kb) will provide a more confident phylogenetic relationship and admixture pattern among these three rice populations.

The results of this study suggest that the weedy rice in Korea originated directly from *japonica/japonica* or *indica/indica* rice hybridization rather than from wild rice. Three major results support this speculation. (i) The phylogenetic tree based on 2.2 million SNPs indicated that 30 weedy rice samples clearly clustered into two major subgroups with *japonica* landrace and *indica* landrace rice, respectively. Weedy rice was more closely related to landrace than wild rice in either the *japonica* group or *indica* group, based on the evolutionary tree topology (Figure 1a). (ii) The results of the ancestry study of 70 samples showed that weedy rice divided into *ind_weedy* and *jap_weedy* subgroups and that landrace rice divided into *ind_landrace* and *jap_landrace* subgroups (Figure 1b). From *K *=* *3 to *K *=* *6, two major weedy rice subgroups (G1, G3) were separated from *indica* landrace and *japonica* landrace rice. Another main weedy subgroup, G2, had many admixture patterns from *jap_landrace* and other *jap_weedy*. There were no wild rice patterns in weedy rice. Therefore, we propose that *ind_weedy* has only two genomic components: *ind_landrace* and *ind_weedy*. It was clearly demonstrated that *jap_weedy* had only two genomic components: *jap_landrace* and *jap_weedy*. (iii) The PCA study showed that weedy rice clearly clustered with landrace rice and not wild rice (Figure 1c).

### Genomic adaptive of weedy rice

Determining the influences of positive and purifying selection, as well as neutral forces in shaping genetic variation is the primary goal of evolutionary biology (Evans *et al*., 2014). Genetic diversity and phenotypic plasticity allow weeds to exploit novel and diverse opportunities as they occur in and infest agroecosystems (Dekker, 1997), there has long been an interest in understanding the genetic basis of weedy rice adaptation (Delouche and Labrada, 2007; He *et al*., 2014; Qiu *et al*., 2014). Understanding the adaptive genomic loci is a prerequisite for designing relevant strategies for effective control and management of different types of weeds in agroecosystems, as well as using these adaptive loci to improve cultivated rice. With the large genome‐wide data sets for 70 rice variations discussed here, there is now an unprecedented opportunity for investigating issues such as the extent to which phenotypic differences among weedy rice and cultivated rice (*O. sativa*) populations are driven by natural selection.

In this study, we concluded that weedy rice formed from cultivated rice. We performed positive selection or adaptive genomic loci identification using five different approaches between weedy and landrace rice. The ROD between two populations was used to detect reduced diversity at putatively neutral sites. *F*_ST_ was used to detect excess SNPs (or regions) with extreme population differentiation. XP‐CLR, which is not sensitive to SNP ascertainment bias, was used to detect the selective sweeps based on multilocus allele frequency differentiation between two populations. XP‐EHH and iHS were used to detect ongoing or nearly fixed selective sweeps by comparing haplotypes from two populations. A total of 537 genes were detected by at least two approaches, and this included many important genes; that is, the abiotic stress‐related genes *SNAC3* on chromosome 1 (Fang *et al*., 2015), *OsAsr1* on chromosome 2 (Vaidyanathan *et al*., 1999), and *OsGIRL1* on chromosome 2 (Park *et al*., 2014); the panicle morphology‐related gene *OsRCN2* on chromosome 2 (Nakagawa *et al*., 2002); biotic stress‐related genes *PDR8* on chromosome 1 (Stein *et al*., 2006) and *Snl6* on chromosome 1 (Bart *et al*., 2010); the ion channel gene related to biotic and abiotic stresses OsCNGC on chromosome 12 (Nawaz *et al*., 2014); the chlorophyll IIb biosynthesis‐related gene *CAO* on chromosome 11 (Oster *et al*., 2000); tiller development‐related gene *SAD1* on chromosome 8 (Li *et al*., 2015); and others (Table S4). It is well known that weedy rice always accompanied by red pericarp, while most cultivated rice have white pericarp, which mainly because of the 14 bp deletion of *Rc* gene on chromosome 7. *Rc* was already proved as the domesticated gene in rice domestication. But we did not find this gene among all the candidate selective genes. This is because we used all SNPs without Indels for selective signature detection. But selective sweeps always clustered or had selective blocks on chromosomes due to the linkage disequilibrium or other reasons. We mapped all 537 genes to the whole rice chromosomes. It is clearly shown that these candidate genes clustered on certain regions of rice chromosomes (Figure S3). When we checked the *Rc* region, two genes (Os07g0200700 and Os07g0222300) were found in 600 kb frank region of *Rc* (Figure S3). Around these two genes may have selective block and genetic linked with *Rc*. There's a big selective block from 8.7 to 13.6 m on chromosome 5 (Figure S3). However, we are did not find the popular domesticated genes in this region. We presume that this region may became hot research region for weedy rice study in the future. Although most of the candidate selection genes have not been cloned and their functions are not yet clear, most of these genes appear to be related to very important functions for weedy rice or cultivated rice. Os01g0191300 (SNAC3) (Fang *et al*., 2015), which was detected using *F*_ST_ and XP‐CLR, was recently confirmed to be related to drought and heat tolerance in rice, providing us a stronger reason to suggest that most of the candidate selected genes played important roles in rice or weedy rice evolution.

We noticed that the genes enriched in response to stress gene ontology terms, with rich factor 0.02 in biological process by using GO analysis (Figure 2b and Table S5). KEGG pathway enrichment suggested roles of these genes in histidine metabolism, which drives the reproduction and plant development‐related pathway (Figure 2c and Table S6). These supported the idea that weedy rice has high reproduction (Song *et al*., 2009) and high stress‐resistant capacity (Chen *et al*., 2004; Liu *et al*., 2014; Lu *et al*., 2014; Qiu *et al*., 2014; Sun *et al*., 2013). Meanwhile there are additional 26 GO terms (*Q *<* *0.05) and 58 pathways were enriched among these 537 genes. It is suggested that the adaptive regions in weedy rice may not only just relate to stress or plant development but also relate to many other biological progress. The tissue‐specific expression pattern study suggested that these candidate selection genes plays different roles at different tissues (Table S9 and Figure S2). The further study based on published databases suggested that most of the genes were related to the plant development and stress resistance. Except 29% unknown genes, others are all related to plant development, abiotic stress and biotic stress among 537 candidate genes (Figure 3a). Among those, 107 genes were enriched in biotic stress pathway, based on MapMan database (Figure 3c). These genes are distributed mostly in the sub‐biological process response to biotic stresses, especially in signalling and proteolysis processes. Moreover, many of the candidate genes are LRR motif enriched gene, receptor‐like cytoplasmatic kinases genes and S‐locus genes (Figure 3d), which are related to plant development, defence and self‐incompatibility based on previous studies (Diévart and Clark, 2004; Vij *et al*., 2008; Xing *et al*., 2013). In summary, these results suggested that weedy rice population significantly accumulated plant development and defence‐related gene variations during the evolution. This may be the main reason why weedy rice exploit novel and diverse opportunities as they occur in and infest agroecosystems.

The results of this study using high‐density polymorphism information clearly showed that Korean weedy rice formed by hybridization of *O. sativa* rather than wild rice. The weedy rice populations contain many genomic adaptive loci, which are different from other populations. These valuable candidate‐selective genes or regions will make weedy rice a good resource to improve cultivated rice in the future, and the total 2.2 million high‐quality SNPs generated here can be used for multiple objectives in the further rice genetics, genomics or evolution studies.

## Experimental procedures

### Plant materials

In 2010, we developed one core set for 4406 worldwide varieties, which collected from the National Genebank of the Rural Development Administration (RDA‐Genebank, Republic of Korea) using the program PowerCore (Kim *et al*., 2007; Zhao *et al*., 2010). Among those, there are 30 Korean landrace rice and 30 Korean weedy rice accessions. These 60 available accessions were maintained by selfing at Kongju National University Experimental Farm. Each accession was transplanted in two rows, with 15 cm between plants and 30 cm between rows. Field management essentially followed normal agricultural practice. Total genomic DNA from a single plant of each accession was extracted from the leaf tissues by using the DNeasy Plant Mini Kit (Qiagen).

### Whole‐genome resequencing, SNP calling and structure variation calling

In previous study, we resequenced 137 core rice accessions (Kim *et al*., 2016). Here, we use 60 of 137 sequence data and ten wild rice sequence data for this study. HiSeq 2500 were used for whole‐genome resequencing of the 60 rice accessions (Kim *et al*., 2016). Raw sequences were first processed to remove residual adapter sequences from the reads using Trimmomatic ver 0.36 (http://www.usadellab.org/cms/index.php?page=trimmomatic). Next, high‐quality reads were aligned to the rice reference genome IRGSP‐1.0 (http://rapdb.dna.affrc.go.jp/download/irgsp1.html) using the Burrows–Wheeler Aligner (BWA) (version 0.7.5a) with the default parameters (Li and Durbin, 2009). The reads will be removed if it did not meet BWA quality criteria or did not align to the reference genome. Duplicate reads were removed by using PICARD (version 1.88) (http://broadinstitute.github.io/picard/). Regional realignment and quality score recalibration were carried out using the Genome Analysis Toolkit (version 2.3.9 Lite) (McKenna *et al*., 2010), and then variations were identified with ≥3× read depth coverage. Overall, the mapping depth was about 10× on average (Table S2). Sequence data for ten wild rice accessions (*O. rufipogon*,* n *=* *5; *O. nivara*,* n *=* *5) were downloaded from the National Centre for Biotechnology Information under accession number of SRA023116, and the same method, used for the 60 accessions, was used for SNP calling. The copy number variation study was performed by CNVnator (Abyzov *et al*., 2011). Inversion and translocation were detected by using DELLY program (Rausch *et al*., 2012).

### Population structure and positive selection analysis

The population phylogenetic tree was constructed by the maximum likelihood method using RaxML (Stamatakis, 2014), and FigTree (Rambaut, 2007) was used to display the tree. We used a maximum likelihood method‐based program, Frappe (Tang *et al*., 2005), to generate the population structure. For positive selection, we used VCFtools (Danecek *et al*., 2011) for nucleotide diversity and *F*_ST_ analysis. We performed XP‐CLR using the XP‐CLR program (http://genepath.med.harvard.edu/~reich). XP‐EHH and iHS were performed by using selscan software (Szpiech and Hernandez, 2014).

### Gene ontology (GO), Kyoto Encyclopedia of Genes and Genomes (KEGG) pathway

All candidate selective genes were used to GO and KEGG analysis. GO enrichment analysis was performed with PlantGSEA (Yi *et al*., 2013) with the ‘Oryza sativa’ set as species background. KEGG pathway was performed by KOBAS (Xie *et al*., 2011)**.**

## Author contributions

K.K.W and P.Y.J supervised the projects. K.K.W and P.Y.J contributed materials and analysis tools. H.Q, K.K.W and P.Y.J designed the research and wrote the manuscript.

## Supporting information


**Figure S1** Different linkage disequilibrium(LD) decay patterns of weedy rice, landrace rice and wild rice.
**Figure S2** Co‐expression patterns of candidate selective genes.
**Figure S3** The distribution of all candidate‐selective genes across rice chromosomes.

**Table S1** Materials used in this study.
**Table S2** Summary of mapping information.
**Table S3** Genes over selection outliers by five different selection metrics.
**Table S4** Candidate selection genes with annotation.

**Table S5** Gene ontology of candidate‐selective genes.

**Table S6** KEGG pathway of candiate‐selective genes.

**Table S7** Biological function of candidate‐selective genes.

**Table S8** High‐quality SNPs used in this study.

**Table S9** Tissue‐specific expression patterns of candidate‐selective genes.
