# Supplementary material for: Population genomics identifies the origin and signatures of selection of Korean weedy rice
Source: Plant Biotechnol J. 2016 Sep 30;15(3):357–66. doi: 10.1111/pbi.12630 (PMC5316921; doi:10.1111/pbi.12630)
Supplement: Supplementary file 1 — Figure S1 Different linkage disequilibrium(LD) decay patterns of weedy rice, landrace rice and wild rice. Figure S2 Co‐expression patterns of candidate selective genes. Figure S3 The distribution of all candidate‐selective genes across rice chromosomes. [file PBI-15-357-s006.docx]

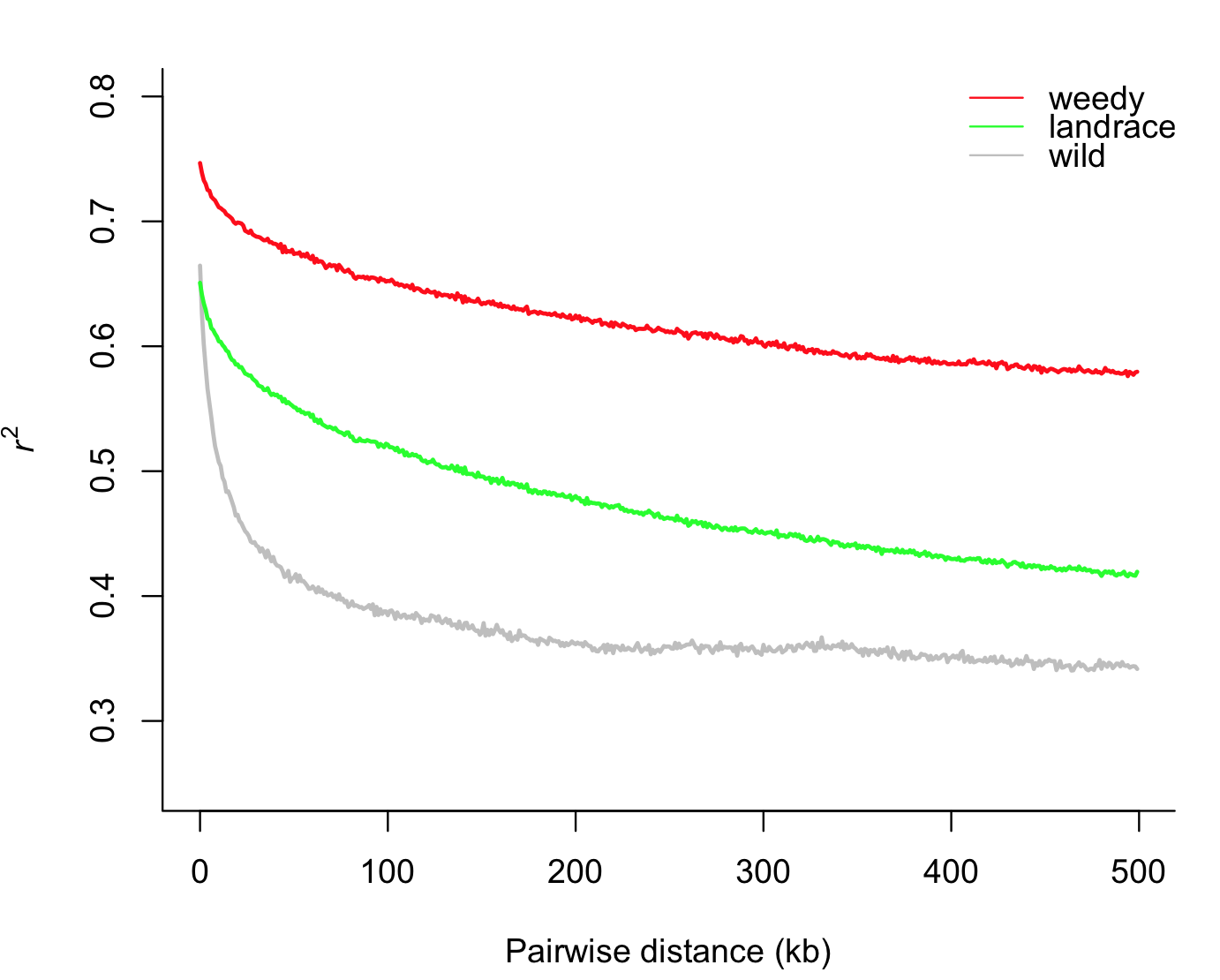


**Figure S1** Different linkage disequilibrium(LD) decay patterns of weedy rice, landrace rice and wild rice.


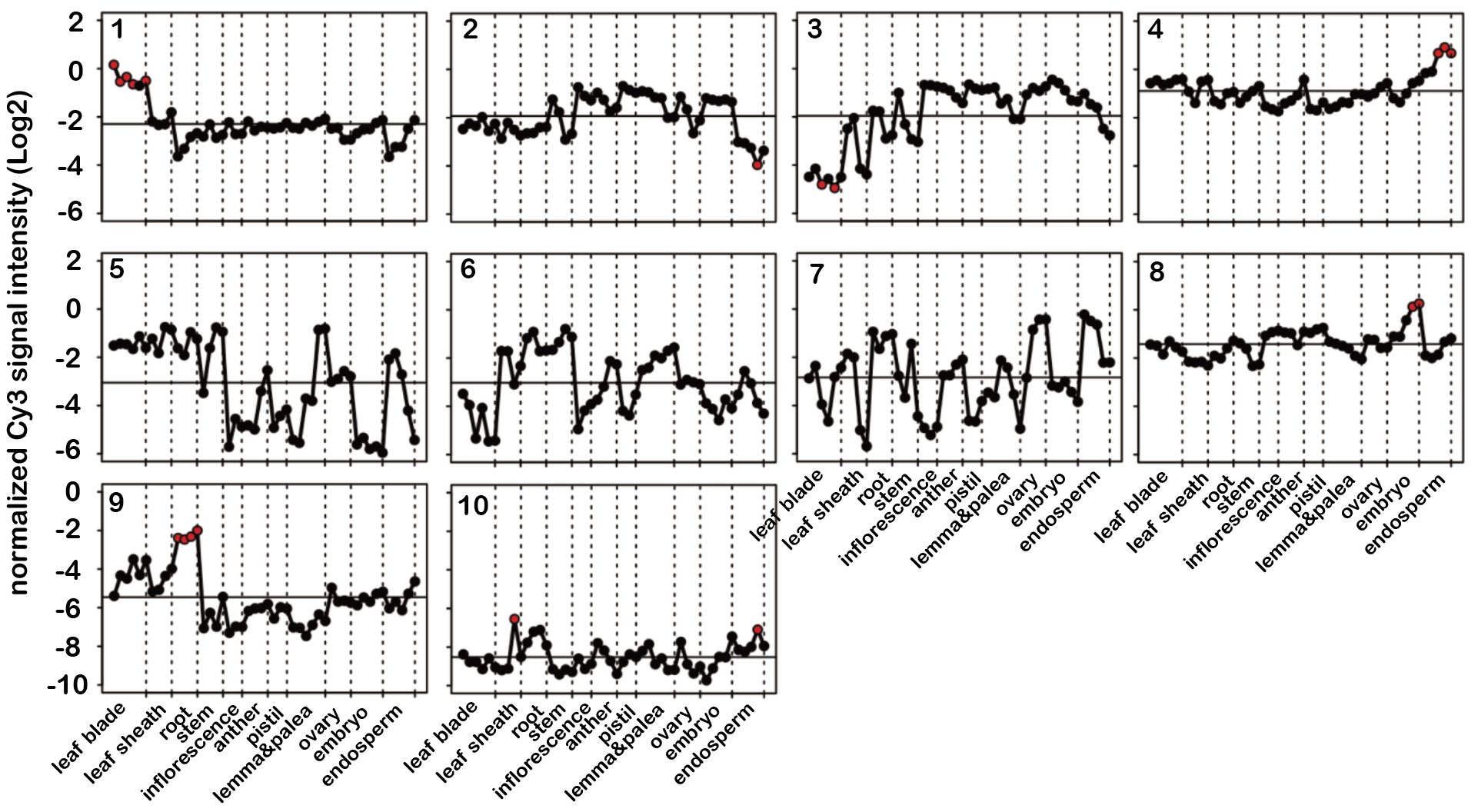


**Supplemental Figure 2.** Co-expression patterns of candidate selective genes. Ten co-expression clusters were detected by *k*-means clustering. Points show the medians of the expression values in different tissue and at different times. Points with expression values deviation over two fold of standard deviation among the cluster are colored in red. The detail rank of points is consistant with Table S9. Horizontal line was average expression value of among the cluster.


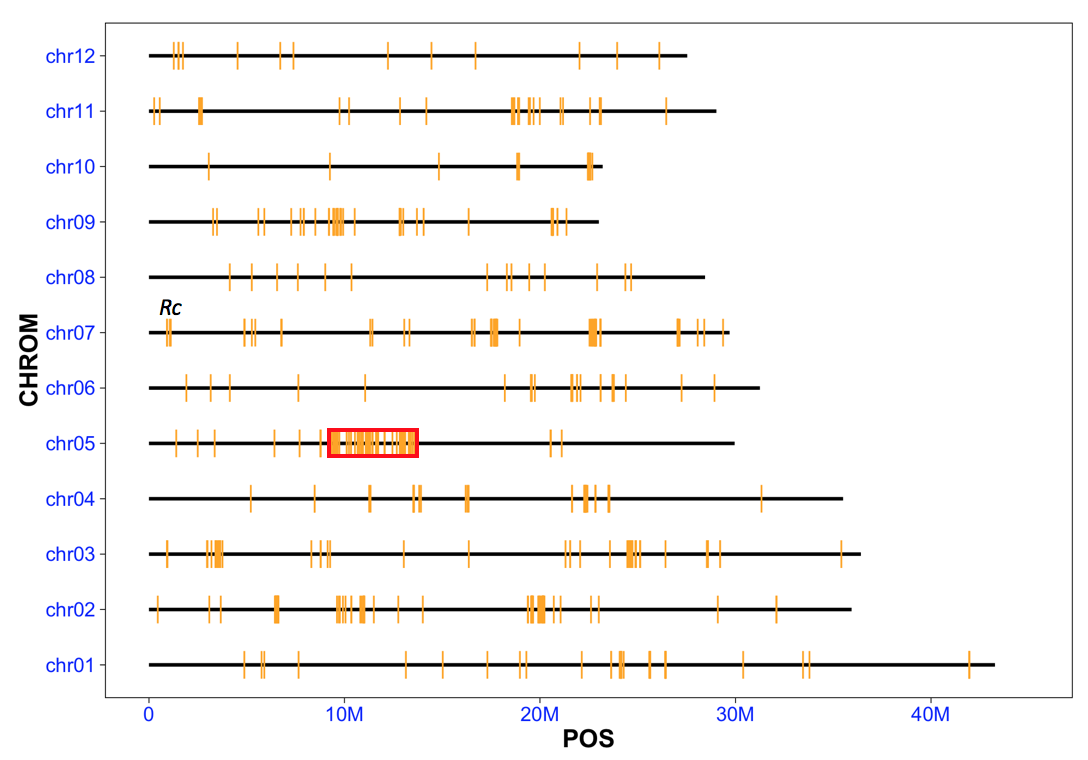


**Supplemental Figure 3.** The distribution of all candidate selective genes across rice chromosomes.
